# Microbiota-host crosstalk in the newborn and adult rumen at single-cell resolution

**DOI:** 10.1186/s12915-022-01490-1

**Published:** 2022-12-14

**Authors:** Jia-Jin Wu, Senlin Zhu, Yi-Fan Tang, Fengfei Gu, Jian-Xin Liu, Hui-Zeng Sun

**Affiliations:** 1 Institute of Dairy Science, College of Animal Sciences, Zhejiang University, Hangzhou, 310058 China; 2 Ministry of Education Innovation Team of Development and Function of Animal Digestive System, Zhejiang University, Hangzhou, 310058 China; 3 Ministry of Education Key laboratory of Molecular Animal Nutrition, Zhejiang University, Hangzhou, 310058 China

**Keywords:** Rumen, Host single-cell transcriptome, Epithelial microbiota, Epithelial metabolome, Microbiota-host crosstalk, *Desulfovibrio*, Pyridoxal

## Abstract

**Background:**

The rumen is the hallmark organ of ruminants, playing a vital role in their nutrition and providing products for humans. In newborn suckling ruminants milk bypasses the rumen, while in adults this first chamber of the forestomach has developed to become the principal site of microbial fermentation of plant fibers. With the advent of single-cell transcriptomics, it is now possible to study the underlying cell composition of rumen tissues and investigate how this relates the development of mutualistic symbiosis between the rumen and its epithelium-attached microbes.

**Results:**

We constructed a comprehensive cell landscape of the rumen epithelium, based on single-cell RNA sequencing of 49,689 high-quality single cells from newborn and adult rumen tissues. Our single-cell analysis identified six immune cell subtypes and seventeen non-immune cell subtypes of the rumen. On performing cross-species analysis of orthologous genes expressed in epithelial cells of cattle rumen and the human stomach and skin, we observed that the species difference overrides any cross-species cell-type similarity. Comparing adult with newborn cattle samples, we found fewer epithelial cell subtypes and more abundant immune cells, dominated by T helper type 17 cells in the rumen tissue of adult cattle. In newborns, there were more fibroblasts and myofibroblasts, an *IGFBP3*^*+*^ epithelial cell subtype not seen in adults, while dendritic cells were the most prevalent immune cell subtype. Metabolism-related functions and the oxidation-reduction process were significantly upregulated in adult rumen epithelial cells. Using 16S rDNA sequencing, fluorescence in situ hybridization, and absolute quantitative real-time PCR, we found that epithelial *Desulfovibrio* was significantly enriched in the adult cattle. Integrating the microbiome and metabolome analysis of rumen tissues revealed a high co-occurrence probability of *Desulfovibrio* with pyridoxal in the adult cattle compared with newborn ones while the scRNA-seq data indicated a stronger ability of pyroxidal binding in the adult rumen epithelial cell subtypes. These findings indicate that *Desulfovibrio* and pyridoxal likely play important roles in maintaining redox balance in the adult rumen.

**Conclusions:**

Our integrated multi-omics analysis provides novel insights into rumen development and function and may facilitate the future precision improvement of rumen function and milk/meat production in cattle.

**Supplementary Information:**

The online version contains supplementary material available at 10.1186/s12915-022-01490-1.

## Background

Ruminants, which were domesticated to provide meat and milk for humans during the Neolithic [1], largely rely on the rumen system to digest human inedible plant mass and transform this into edible protein [2–5]. Newborn (NB) ruminants are not able to chew the cud and depend on milk-based diets. They are considered functionally monogastric animals due to the underdeveloped rumen and less abundant resident microbiota [6]. In adult (AD) ruminants, the rumen displays a distinct morphological structure and has essential biological functions. For example, the rumen expands gradually after birth to make up more than 70% of total digestive tract volume for efficiently digesting forage-based diets [7, 8]. A previous study also indicated that the rumen was the organ that had the most significant transcriptional differences between newborn and adult stages by comprehensively characterizing transcription of the entire gastrointestinal tract [9]. The rumen is colonized with a complex and diverse community of microbiota. A recent study reported that the ruminal transcription of genes related to the “respiratory electron transport chain” was positively correlated with age as well as with the relative abundance of ruminal bacterial genera [10]. However, to date, there have been few studies explored the development of mutualistic symbiosis between rumen and its microbial colonizers.

The microbiota in the rumen has been classified into three major groups: microbiota in solid digesta, microbiota free-floating in the liquid fraction, and the mucosal microbiota. The microbiota in solid digesta and free-floating in the liquid fraction has been the focal point of the rumen physiology research, whereas the mucosal microbiota has received less attention [11]. Previous studies suggested that the analysis of rumen tissue-attached bacteria may provide a better understanding of ruminant metabolism and host-microbe interactions [11, 12]. To date, however, the underlying interactions between rumen mucosal microbiota and host cells remain largely unknown. It is known that almost the entire rumen epithelium is covered by microbes [13, 14] and that colonization by ruminal epithelial bacteria is age-related [15]. In this respect, the rumen is also a useful model of how microbe-host interactions develop.

Nutrient absorption and microbial interaction are facilitated by the large surface area of the rumen, which is lined with abundant papillae of keratinized stratified squamous epithelium composed of four different cellular strata: basale, spinosum, granulosum, and corneum [16]. Recent advances in single-cell RNA sequencing (scRNA-seq) have paved the way for a more granular analysis of cell types, and the finding that at least 15 cell types can be detected in the intestinal columnar epithelium of humans [17] led to speculation that more discrete epithelial cell populations exist in the rumen [18]. In this study, we have taken a multi-omic approach that includes scRNA-seq to explore the cell-type composition, associated microbiome, and metabolome of rumen epithelium in a comparative analysis of undeveloped rumen tissues from newborn calves and mature rumen tissues from adult lactating cattle. Our scRNA-seq data from the rumens of these adult cattle has also been reported and analyzed in two other studies (embarked upon after this one, but already published [19, 20]).

In one study [19], we compared scRNA-seq data and metabolites from multiple tissue types (rumen, reticulum, omasum, abomasum, ileum, rectum, liver, salivary gland, mammary gland, and peripheral blood), originating from the same lactating adult dairy cattle. In the other study [20], we investigated the contribution of interactions between adult rumen epithelial cell subtypes and rumen digesta microbiota to fiber utilization by integrating the data of meta-genome assembled microbial genomes and scRNA-seq [20]. In the current study, we used scRNA-seq for analyzing cell-type/subtype composition and function of NB and AD rumen in dairy cattle and performed the cross-species analysis between the cattle rumen and the human stomach and skin. 16S rRNA gene sequencing was conducted to analyze the mucosal bacterial composition. To gain insights into how the mucosal bacteria interact with the host’s cells, we performed metabolomics of NB and AD rumen tissues. Integration of epithelial microbiome, metabolome, and single-cell transcriptome was further explored to infer the microbiota-rumen cell-type interactions.

## Results

### Single-cell atlas of the rumen in NB and AD cattle

**Fig. 1:**
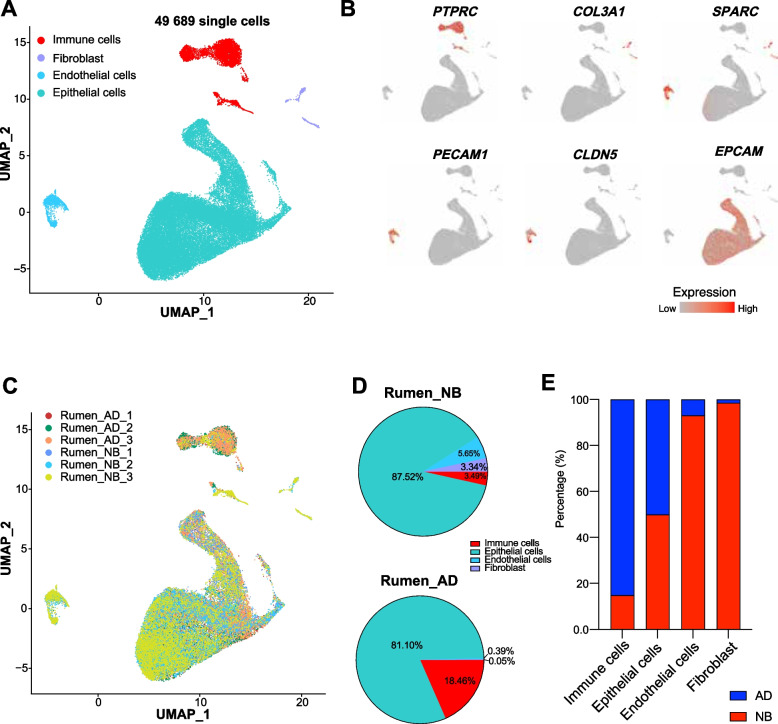
Dissection of the ruminal cell type composition with scRNA-seq in NB and AD dairy cattle. **A** UMAP plots of cells from the 6 samples profiled in this study, with each cell color coded to indicate the associated cell types. **B** The UMAP plots show the expression of curated genes in the cell types defined. **C** The UMAP maps of adult (AD) and newborn (NB) rumen single-cell data, with each cell color coded to indicate the associated samples. **D** Pie chart showing the relative percentages of different major cell types in newborn calves and adult cattle. **E** The cell-type contribution bar charts show the distribution of major cell types in NB and AD rumen samples. NB: newborn; AD: adult

To delineate the cell-type composition dynamics of the rumen between NB and AD stages, we generated scRNA-seq profiles for rumen tissues from three newborn calves and three adult dairy cattle. After quality filtering, we profiled a total of 49,689 high-quality single cells and classified the cells into coherent transcriptional clusters (Fig. 1A). Next, we annotated the cell clusters by the average expression of canonical marker genes and identified four major cell types: immune cells (high *PTPRC* expression), fibroblast (high *COL3A1* and *SPARC* expression) [21], endothelial cells (high *PECAM1* and *CLDN5* expression) [22], and epithelial cells (high *EPCAM* expression) [23] (Fig. 1A, B). Multi-donor analysis of rumen tissues showed limited effects on cell-type discovery in NB or AD tissues (Fig. 1C). Epithelial cells were the most abundant cell type in both NB and AD groups, accounting for 87.52 and 81.10% of total cells, respectively (Fig. 1D). Immune cells were mainly identified from adult cattle, while most endothelial cells and fibroblasts were from newborn calves (Fig. 1E). The number of the four major cell types was also available in Table S1. There are many subtypes of immune cells and non-immune cells; however, little is known about them in the rumen. We next analyzed the rumen immune cells and non-immune cells separately (see “Methods”).

### Immune cell subtypes and difference between NB and AD cattle

**Fig. 2:**
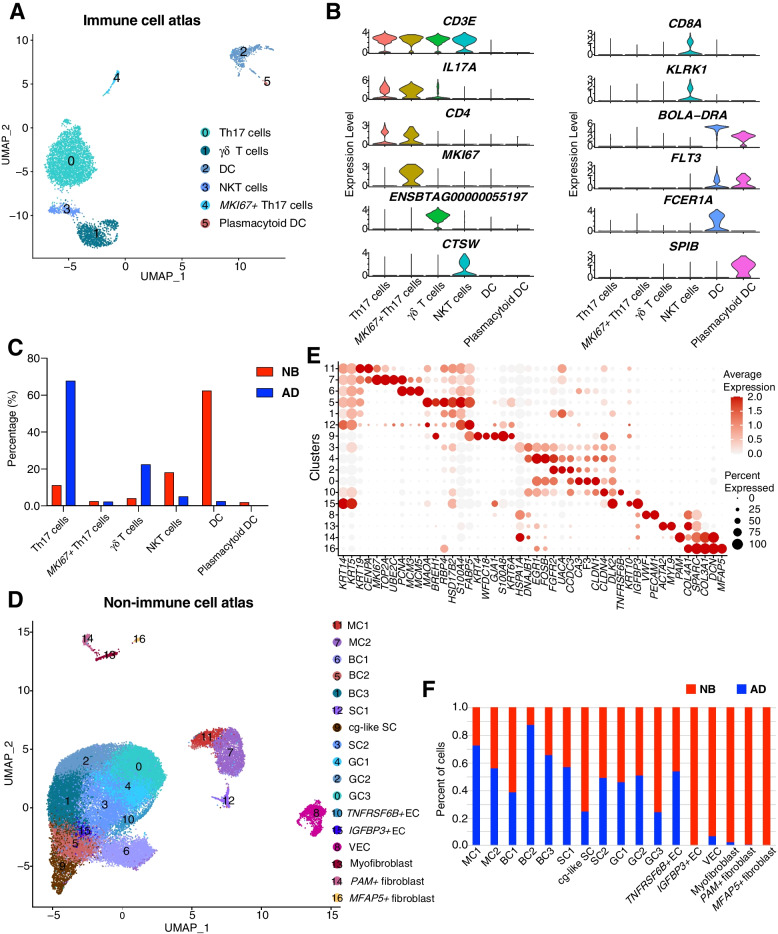
Dissection of the immune and non-immune cell composition in NB and AD rumen tissues. **A** UMAP plots of immune cells, with each cell color coded to indicate the associated cell types. **B** The violin plots showing gene expression of markers of each immune cell types. **C** Bar graph showing the relative percentages of different immune cell types in newborn calves and adult cattle. **D** UMAP plots of non-immune cells, with each cell color coded to indicate the associated cell types. **E** The dot plot visualization of each cell type in rumen non-immune cell data. Dot size represents the percentage of cells within a cell type, and the color encodes the expression level. **F** The cell-type contribution bar charts show the distribution of cell types in NB and AD rumen samples. NB: newborn; AD: adult. NKT: natural killer T cells, DC: dendritic cells; MC1: mitotic cell 1; MC2: mitotic cell 2; BC1: basal cell 1; BC2: basal cell 2; BC3: basal cell 3; SC1: spinous cell 1; SC2: spinous cell 2; cg-like SC: channel-gap-like spinous cell; GC1: granule cell 1; GC2: granule cell 2; GC3: granule cell 3; *TNFRSF6B*^*+*^ EC: *TNFRSF6B*^*+*^ epithelial cell; *IGFBP3*^*+*^ EC: *IGFBP3*^*+*^ epithelial cell; VEC: vascular endothelial cell

In total, immune cells were clustered into six separate subtypes (Fig. 2A): Th17 cells, *MKI67*^*+*^ Th17 cells, γδ T cells, natural killer T (NKT) cells, dendritic cells (DC), and plasmacytoid DC. Th17 cells (Cluster 0) expressed the classic markers, including *CD3E*, *CD4*, *IL17A*, and *IL17F* [24] (Fig. 2B; Additional file 2: Table S2). Except for the markers of Th17 cells, Cluster 4 uniquely expressed *MKI67* and was annotated as *MKI67*^*+*^ Th17 cells (Fig. 2B). In humans, γδ T cells usually present a *CD3E*^*+*^*CD4*^*-*^*CD8A*^*-*^ cell surface phenotype and express the TCRδ constant region-encoding segment *TRDC* [25]. Cluster 1 showed high expression of *CD3E* and a bovine homolog of *TRDC* but no expression of *CD4* and *CD8A*, which suggested that these cells were γδ T cells (Fig. 2B; Additional file 2: Table S2). Cluster 3 was defined as NKT cells due to its specific expression of *CD3E*, *CTSW*, *KLRK1*, *NKG7*, *CCL5*, and *CD8A* [26, 27] (Fig. 2B; Additional file 2: Table S2). Cluster 2 was DC for highly expressing *FLT3*, *CTS3*, and *BOLA-DRA* as well as the conventional type 2 dendritic cell marker genes *FCER1A* and *CD1E*, and Cluster 5 was believed to represent plasmacytoid DC that expressed *SPIB*, *BLNK*, and *ITM2C* [28, 29] (Fig. 2B; Additional file 2: Table S2). Our other paper, which analyzed the same scRNA-seq data form the AD rumen but without the NB rumen, also identified the Th17 cells, *MKI67*^*+*^ Th17 cells, γδ T cells, and DC, but no NKT cells and plasmacytoid DC [19]. However, we found the NKT cells and plasmacytoid DC by integrating scRNA-seq datasets of the NB and AD rumen in this study. The reason for the inconsistency is that when analyzing solely the scRNA-seq data of the AD rumen, the NKT cells and plasmacytoid DC were algorithmically grouped with a more populous cell type due to the small number (see below). This is a predictable artefact of the annotation scheme: entire cell clusters, rather than individual cells, were annotated in each tissue. Th17 cells represented the most prevalent immune cell type in the AD rumen, while DC was the prevalent immune cell type in NB rumen. Compared with NB calves, the average percentage of Th17 cells (67.83% vs. 11.13%) and γδ T cells (22.41% vs. 4.02%) were increased in the AD cattle, while the average percentages of DC (2.40% vs. 62.44%), plasmacytoid DC (0.06% vs. 1.88%), and NKT (5.05% vs. 18.11%) cells were decreased in the AD cattle (Fig. 2C). These results provide novel insights into the different rumen immune microenvironment between NB and AD stages.

### Non-immune cell subtypes and cross-species comparison

In addition to immune cell subtypes, we observed 17 cell clusters of non-immune cells including 13 subtypes of rumen epithelial cells (EC), 3 fibroblast subtypes, and 1 vascular endothelial cell (VEC) subtype based on the feature plots of known cell-type markers (Figs. 2D, E; Additional file 2: Table S3). Basal cells (BC), spinous cells (SC), and granule cells (GC) are the living epithelial cell types currently characterized in the rumen [30, 31]. Three BC (high *KRT14* and *KRT5* expression) [32–34], three SC (high *S100A8* or *KRT6A* expression) [35], and three GC (high *CLDN4*, *CLDN1*, or *DLK2* expression) [30, 31, 36] cell subtypes were discovered in the rumen (Fig. 2D, E). In addition, we revealed two mitotic cell (MC) subtypes based on the expression of the marker genes of *KRT14* and *MKI67* [34, 37] (Fig. 2E). The mitotic cell 1 (MC1, Cluster 11) also highly expressed *KRT19*, *CENPA*, *CENPF*, etc., and the mitotic cell 2 (MC2, Cluster 7) also highly expressed *TOP2A*, *UBE2C*, etc. (Fig. 2E; Additional file 2: Table S3). Cluster 9 spinous cell subtype specifically highly expressed *GJA1*, encoding a channel gap junction protein [38]; thus, it was annotated as channel-gap-like spinous cells (cg-like SC). Interestingly, Cluster 10 and Cluster 15 commonly expressed BC and GC markers but uniquely expressed *TNFRSF6B* and *IGFBP3*, respectively. Thus, Cluster 10 and Cluster 15 were defined as *TNFRSF6B*^*+*^ EC and *IGFBP3*^*+*^ EC, respectively. In our other paper [20], we also found the MC, BC, cg-like SC, SC, and GC cell types and all of them had more subtypes than that of this study (3 vs. 2, 4 vs. 3, 3 vs. 1, 3 vs. 2, and 5 vs. 3, respectively). This was in line with expectations because our other paper [20] carried out reclustering analysis on the epithelial cells collected from the same scRNA-seq data of AD rumen [19] with higher resolution (1.6 vs. 0.8), which can identify more cell subtype clusters. It should be noted that the *TNFRSF6B*^*+*^ EC was observed in this study but not in our other study. Given that the GC_5 cell cluster identified in our other study uniquely highly expressed the *TNFRSF6B* gene but not BC marker genes [20], the *TNFRSF6B*^*+*^ EC cell cluster may be a combination of GC_5 and basal cells in this study. This suggests that it may be necessary to perform the reclustering analysis with higher resolution on the epithelial cells identified in this study in the future.

Diverse rumen structural cell subtypes were identified, including VEC (Cluster 8), myofibroblasts (Cluster 13), *PAM*^*+*^ fibroblast (Cluster 14), and *MFAP5*^*+*^ fibroblast (Cluster 16) (Fig. 2D, E). A list of differentially expressed genes for each cluster was also available in Table S3. In our other paper [19], the myofibroblasts, *PAM*^*+*^ fibroblast, and *MFAP5*^*+*^ fibroblast cell clusters were not identified. This is because they could not form the cell cluster and algorithmically grouped with a more populous cell cluster due to the small number (only 9, 2, and 0, for the myofibroblasts, *PAM*^*+*^ fibroblast, and *MFAP5*^*+*^ fibroblast cell clusters, respectively; Additional file 2: Table S1) when analyzing solely the scRNA-seq data of the AD rumen. The results suggest that a sufficient number of cells are needed to identify a cell type in the single-cell RNA sequencing studies.

The rumen is the hallmark organ of ruminants, which is likely to have different cell composition from human and other monogastric animal stomach or other organs. To compare the cattle rumen and the human cell landscape, we downloaded a human stomach single-cell RNA-seq dataset from a previous study [23] and performed cell clustering analysis. We identified 23 cell clusters in the human stomach and identified 8 epithelial cell clusters based on highly expressed marker genes, such as *EPCAM*, *KRT19*, *KRT18*, *KRT8*, *PGA5*, *CKN1*, *CHGA* (Additional file 1: Figs. S2A and B; Additional file 2: Table S4). Considering that circulating immune cells that derive from the same lineage are widely distributed within the mammalian body, the current study focused on the cross-species comparison of epithelial cells (see “Methods”). A correlation heatmap among 13 rumen and 8 human stomach epithelial cell clusters showed that the similarities of epithelial cells between the two species were very low; all the epithelial cell subtypes of the cattle rumen have a weak correlation with the cell clusters of the human stomach (area under the receiver operating characteristics (AUROC) score < 0.9) (Additional file 1: Fig. S2C).

Similar to the rumen tissue, the human skin tissue is also composed of stratified squamous epithelium [38] and shared a significant number of transcriptomic features and conserved gene expression patterns with the rumen [39]. Therefore, we also downloaded the human skin single-cell RNA-seq dataset from a previous study [37] and performed cell clustering analysis. In the human skin, we identified 17 epithelial cell clusters based on highly expressed marker genes, such as *KRT14*, *KRT5*, *KRT15*, *KRT19*, *KRT18*, *KRT6A*, *KRT10*, *KRT1*, and *FLG* (Additional file 1: Figs. S3A and B; Additional file 2: Table S5). By performing cross-species analysis of 13 rumen and 17 human skin epithelial cell clusters (see “Methods”), we found that the gene expression patterns of most epithelial cell clusters were not conserved (AUROC score < 0.9); only 3 epithelial cell clusters of human skin had a strong correlation with rumen cell clusters (AUROC score > 0.9) (Additional file 1: Fig. S3C). Cluster 10 in the human skin had a strong correlation with cg-like SC of the rumen (Additional file 1: Fig. S3C) and expressed some marker genes of cg-like SC, such as *GJA1*, *FABP5*, and *KRTDAP* (Additional file 2: Table S5). Despite this, only 18 of the top 100 marker genes were shared between the two cell clusters (Additional file 2: Tables S3 and S5), and other genes of the top 100 genes in cattle that were different from humans were enriched in the “fatty acid binding” (such as *S100A8*, *S100A9*, and *FABP4* genes) and “fatty acid derivative metabolic process” (such as *GSTA1*, *MGST3*, and *ATP6V1B1* genes) based on the Gene Ontology (GO) enrichment analysis, which may benefit cattle’s energy acquisition, as fatty acids, rather than glucose, are the main source of energy in ruminants. Cluster 18 in the human skin had strong correlations with the MC1 and MC2 of the rumen; and Cluster 11 in the human skin had a strong correlation with the MC2 (Additional file 1: Fig. S3C). Clusters 18 and 11 in the human skin also highly expressed some marker genes of MCs, such as *MKI67*, *CENPF*, and *HMGB2* (Additional file 2: Table S5). However, only 38 and 50 of the top 100 marker genes of MC1 and MC2 were shared with that of Cluster 18 in the human skin, respectively, and only 35 of the top 100 marker genes were shared between the cattle MC2 and Cluster 11 of the human skin (Additional file 2: Table S3 and S5). Taken together, our findings suggested that almost all epithelial cell types in the rumen of cattle and the stomach and skin of humans were not similar.

Although newborn calves are considered functionally monogastric animals due to the underdeveloped rumen [6], it is interesting to note that almost all the rumen epithelial cell subtypes were presented in both NB and AD cattle, except for *IGFBP3*^*+*^ EC, which were only identified in NB calves (Fig. 2F). The myofibroblast and *PAM*^*+*^ fibroblasts were predominantly distributed in the NB calves, and the *MFAP5*^*+*^ fibroblasts were not found in the AD cattle in this study (Fig. 2F). To some extent, the dissociation bias inherent in all solid tissues single-cell experiments may cause spurious changes in cell numbers. However, through reanalysis of previously published transcriptome data obtained by the bulk RNA-seq experiment from newborn [10] and adult [40] cattle rumen tissues, we observed that high expression levels of the fibroblast marker genes *COL3A1*(9,230 vs. 636.8, counts per million (CPM)), *PAM* (293.3 vs. 148.8, CPM), and *MFAP5* (173.8 vs. 7.6, CPM) in the newborn rumen tissues whereas very low expression levels in adult rumen tissues (Additional file 1: Fig. S1).

### Functional differences of cell types between NB and AD cattle

**Fig. 3:**
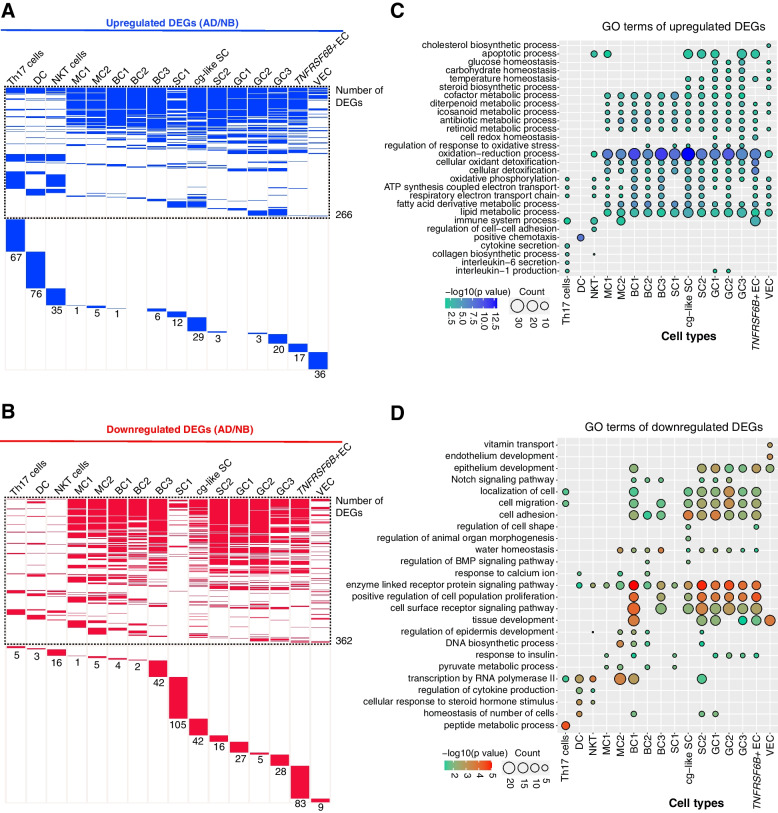
Changes in the transcriptional profiles of different cell types between NB and AD rumen. **A, B** Heatmaps show the distribution of upregulated (**A**, blue) and downregulated (**B**, red). The upper part (dotted lines) represents the DEGs shared by at least two cell types, and the lower panel represents the unique DEGs of each cell type, and the number below the doted box represents the number of the unique DEGs in each cell type. **C, D** The representative GO terms of cell types (enriched in upregulated and downregulated DEGs, respectively; *p*-value < 0.05). NB: newborn; AD: adult. NKT: natural killer T cells, DC: dendritic cells; MC1: mitotic cell 1; MC2: mitotic cell 2; BC1: basal cell 1; BC2: basal cell 2; BC3: basal cell 3; SC1: spinous cell 1; SC2: spinous cell 2; cg-like SC: channel-gap-like spinous cell; GC1: granule cell 1; GC2: granule cell 2; GC3: granule cell 3; *TNFRSF6B*^*+*^ EC: *TNFRSF6B*^*+*^ epithelial cell; VEC: vascular endothelial cell

In addition to cell-type composition heterogeneity, we further deciphered the functional differences in the specific pairs of cell types between NB and AD rumen. We first divided the dataset into NB and AD groups and compared gene expression patterns of individual cell types between groups (see “Methods”). Compared to the NB group, we identified 266 upregulated and 362 downregulated differentially expressed genes (DEGs) (|log_2_FC| > 0.5, adjusted *p*-value < 0.05), both of which were shared by at least two cell types (Fig. 3A, B; Additional file 2: Table S6). We also identified the cell-type-specific upregulated and downregulated DEGs (Fig. 3A, B; Additional file 2: Table S6). Interestingly, we note that the immune cell subtypes had a larger number of cell-type-specific upregulated DEGs than cell-type-specific downregulated DEGs whereas the epithelial cell subtypes had fewer cell-type-specific upregulated DEGs than cell-type-specific downregulated DEGs in the AD cattle.

To further explore which biological processes the DEGs are involved in, we performed GO enrichment analysis of up- and downregulated DEGs. The upregulated DEGs across all the rumen epithelial cell subtypes in AD cattle were mainly enriched in GO terms of the “fatty acid derivative metabolic process,” “oxidation-reduction process,” and the metabolic processes of retinoid, antibiotic, icosanoid, diterpenoid, and cofactor (Fig. 3C), suggesting dramatic shifts in physiology and metabolism of rumen between NB and AD cattle. However, the downregulated DEGs involved GO terms were not consistent among different cell types (Fig. 3D). For instance, the downregulated DEGs in cg-like SC were specifically enriched in the “regulation of animal organ morphogenesis.” The GO term “regulation of epidermis development” was only enriched in the MC1, basal cell 1 (BC1), basal cell 2 (BC2), and spinous cell 2 (SC2). Nevertheless, GO analysis showed that downregulated genes were mainly related to epithelial cell proliferation and receptor signaling pathways across many epithelial cell subtypes. Taken together, these results indicate a series of cell-type-specific molecular changes featuring cell proliferation and development at NB stage, and oxidation-reduction and nutrient metabolism at AD stage in the rumen tissues.

### Rumen mucosal microbial composition difference between NB and AD cattle

**Fig. 4:**
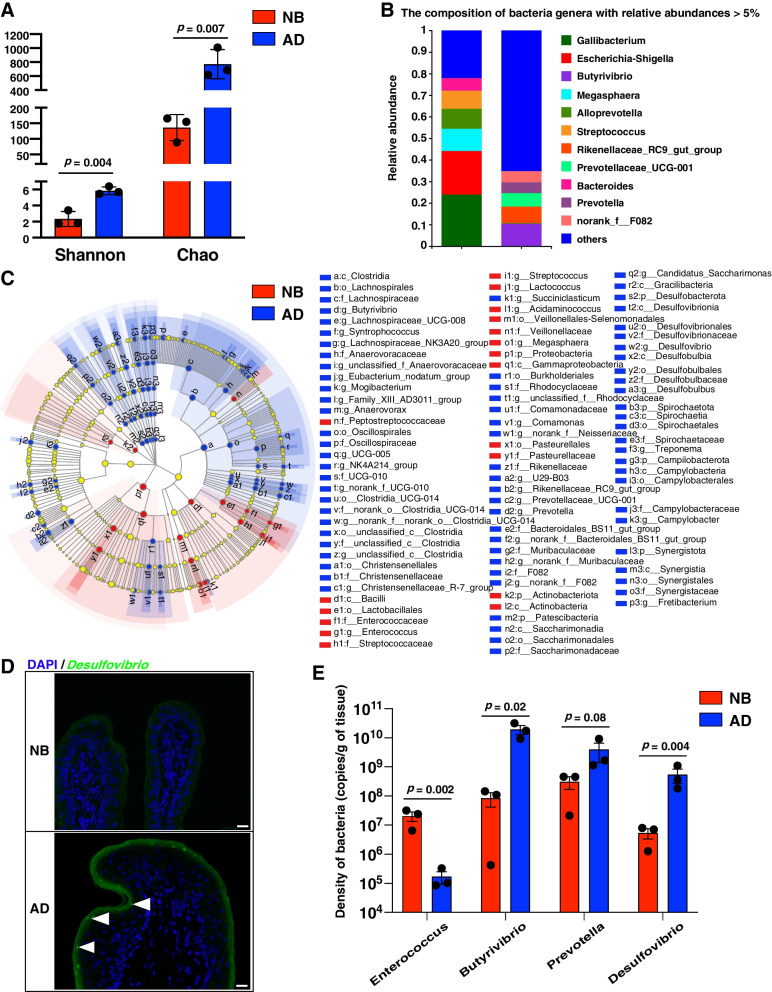
Epithelial microbiota in the rumen tissue between NB and AD dairy cattle. **A** The difference of α-diversity indexes of ASVs between NB and AD groups. **B** The epithelial bacterial composition in NB and AD groups on genera level (relative abundances > 5%). **C** The linear discriminant analysis effect size (LEfSe) analysis and cladogram representation show that bacterial taxa differed significantly different between NB and AD groups. **D** The sections were stained with DAPI (epithelium, blue) and bacterial FISH probe (green) targeting *Desulfovibrio* demonstrating its higher abundance in AD than NB rumen tissues. **E** Differences of rumen epithelial bacteria density between NB and AD groups. NB: newborn; AD: adult

Mucosal microbiota lives in close contact with host cells of rumen tissues, which can execute coordinated functions contributing to the diverse physiological processes of the rumen [41]. To further explore how mucosal microbiota interact with host cells in the newborn and adult rumen, we performed 16S rRNA gene sequencing analysis of the same rumen tissues from NB and AD cattle. To minimize the effects of sequencing depth on alpha diversity measure, the number of sequences from each sample was rarefied to 25,349, which yielded an average Good’s coverage of over 99%. Rarefaction curves approximately trended to a plateau and sequencing coverage was saturated at 15,000 reads (Additional file 1: Fig. S4). We found higher bacterial diversity (2.32 vs. 5.85, *P* = 0.004) and richness (136.7 vs. 770.3, *P* = 0.007) in the AD rumen compared with the NB group (Fig. 4A), which is consistent with the previous studies [15, 42]. The composition of rumen bacteria at the genus level also showed clear discrimination between the two stages (Fig. 4B). Furthermore, a total of 94 significantly different (LDA > 3.5, *P* < 0.05) bacterial taxa were identified between NB and AD groups (Fig. 4C). The NB group was only enriched with 5 bacteria genera (Fig. 4C; Additional file 1: Fig. S5), including the *Megasphaera*, *Enterococcus*, *Acidaminococcus*, *Streptococcus*, and *Lactococcus*. Compared with NB, the AD group was significantly enriched in 32 bacteria genera (Fig. 4C; Additional file 1: Fig. S5), including *Butyrivibrio* and *Prevotella*, which are known to produce SCFAs [15, 43]. The *Desulfovibrio* genus also had a higher relative abundance (1.78% vs. 0.01%) in the AD rumen (Fig. 4C), and the *Desulfovibrio* was also verified by the bacterial fluorescence in situ hybridization (FISH) (Fig. 4D). We further determined the bacterial densities for the *Desulfovibrio* genus and other genera (including *Enterococcus*, *Butyrivibrio*, and *Prevotella*) being differentially abundant between NB and AD groups using absolute quantitative real-time PCR. The bacterial densities of *Desulfovibrio* genus and *Butyrivibrio* genus in AD group were significantly higher than that in NB group (*P* < 0.01) (Fig. 4E). The bacterial density of *Prevotella* genus in AD group was tended to be higher than that in NB group (*P* = 0.08) (Fig. 4E). The bacterial density of *Enterococcus* genus in NB group was significantly higher than that in AD group (*P* < 0.01) (Fig. 4E).

### Metabolome profiles of rumen epithelial tissue in NB and AD animals

**Fig. 5:**
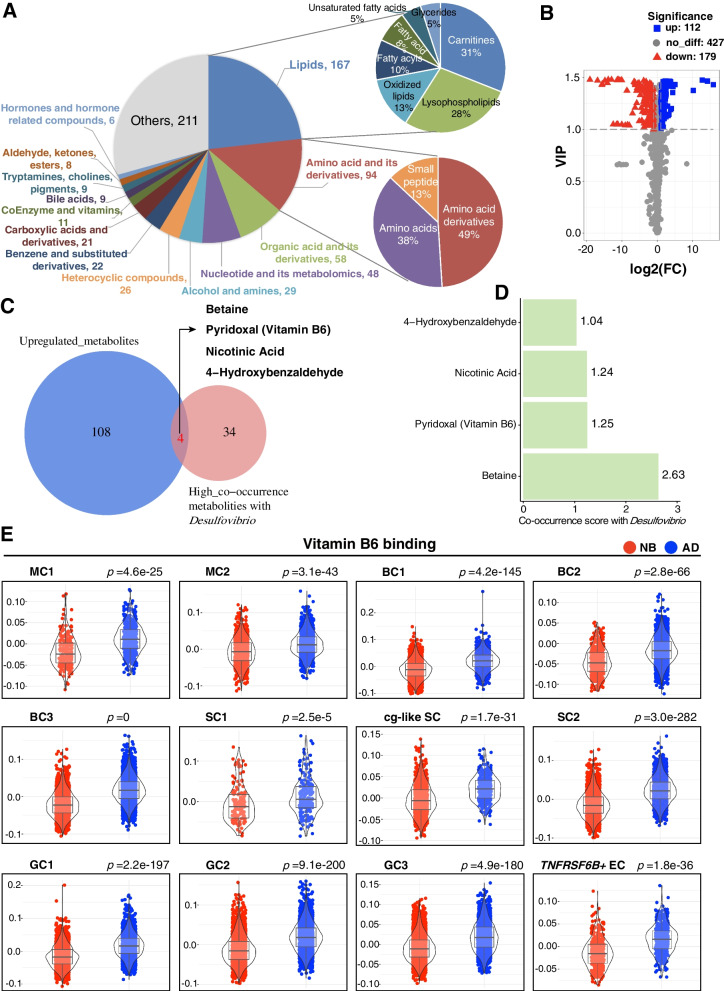
*Desulfovibrio* associated metabolites and cell types in the rumen. **A** The classification of metabolites identified in the rumen tissue. **B** The differential metabolites between NB and AD groups. **C** The Venn diagram represents the overlaps of the upregulated metabolites and the microbiota-dependent metabolites that have high co-occurrence probabilities with *Desulfovibrio*. **D** The co-occurrence probabilities of the four metabolites. **E** Gene scoring analysis of rumen epithelial cells between NB and AD groups using the vitamin B6 binding gene set. *p* values are calculated from two-sided Wilcoxon rank-sum tests. NB: newborn; AD: adult. MC1: mitotic cell 1; MC2: mitotic cell 2; BC1: basal cell 1; BC2: basal cell 2; BC3: basal cell 3; SC1: spinous cell 1; SC2: spinous cell 2; cg-like SC: channel-gap-like spinous cell; GC1: granule cell 1; GC2: granule cell 2; GC3: granule cell 3; *TNFRSF6B*^*+*^ EC: *TNFRSF6B*^*+*^ epithelial cell

In the above sections, we revealed that all the rumen epithelial cell types exerted a stronger function of oxidoreductase activity, and the *Desulfovibrio* had higher relative abundance at AD rumen tissues compared to NB group, which could take part in the host oxidation-reduction process. In many ecosystems, the bacteria play a significant role as manifested by their dynamic interactions with the host. The microbe-host interactions are mediated by the interplay of various molecular components that are expressed by the host and the bacteria. Therefore, we performed a metabolomics analysis in the NB and AD rumen tissues (see “Methods”). In total, 718 metabolites were detected (Fig. 5A), which belong to lipids (*n* = 167), amino acids and their derivatives (*n* = 94), organic acid and its derivatives (*n* = 58), nucleotide and its derivatives (*n* = 48), alcohol and amines (*n* = 29), heterocyclic compounds (*n* = 26), benzene and substituted derivatives (*n* = 22), carboxylic acids and derivatives (*n* = 21), coenzyme and vitamins (*n* = 11), bile acids (*n* = 9), tryptamines, choline, pigments (*n* = 8), hormones and hormone-related compounds (*n* = 6), and other unnamed metabolites (*n* = 211). Among the 167 lipids, 31, 28, 13, 10, 8, 5, and 5% were carnitines, lysophospholipids, oxidized lipids, fatty acyls, fatty acids, unsaturated fatty acids, and glycerides, respectively (Fig. 5A). Among the 94 amino acids and their derivatives, amino acid derivatives, amino acids, and small peptides accounted for 49, 38, and 13%, respectively (Fig. 5A). A total of 112 metabolites were significantly higher (variable importance in projection (VIP) ≥ 1, |Log_2_FC| ≥ 1) while 179 were significantly decreased (VIP ≥ 1, |Log_2_FC| ≥ 1) in the AD group when compared with the NB group (Fig. 5B; Additional file 2: Table S7).

### Desulfovibrio interacts with rumen epithelial cells via pyridoxal

The production of small molecules is an essential route of rumen bacteria influencing host physiology [44, 45], yet it is difficult to accurately monitor the diversity of molecules produced by mucosal bacteria. To address this gap, we used the gut microbial metabolites database [46] and the microbe–metabolite vectors (mmvec) neural networks analysis [47] to identify host-microbe interactions by calculating the co-occurrence probabilities between mucosal bacteria and the ruminal tissue metabolites (see “Methods”). We found that 142 metabolites had high co-occurrence probabilities (the inferred conditional probabilities > 1) with *Desulfovibrio* genera, and 38 of them are microbiota-dependent metabolites against a reference library of microbiota-dependent metabolites [46] (Additional file 2: Table S8). The Venn diagram represents the overlaps of these 38 microbiota-dependent metabolites and the upregulated metabolites, which included betaine, pyridoxal, nicotinic acid, and 4-hydroxybenzaldehyde (Fig. 5C, D). As one form of vitamin B6, pyridoxal is a coenzyme participating in many metabolic reactions, including epithelium proliferation, transportation of amino acids, and inflammatory response [48–50]. And pyridoxal, as an electron donor for sulfate reduction, can be oxidized by *Desulfovibri*o [51], contributing to reducing reactive oxygen species (ROS).

Finally, we computed molecular signature scores of the vitamin B6 binding of rumen epithelial cell types between the two groups using the gene set scoring analysis. The results showed that rumen epithelial cell types scored significantly higher (*P* < 0.05) for vitamin B6 binding in AD group compared with the NB group (Fig. 5E), which implies that the pyridoxal may be implicated in the interaction between the *Desulfovibrio* and the rumen cell types. Further study to unveil the underlying mechanism is needed.

## Discussion

Rumen epithelial microbiota are directly associated with host tissue; thus, it is likely that they play a vital role in mucosal immune systems and ruminant metabolism [12]. Although the last decades have given further insight into the composition of the rumen epithelial microbiota [11], there is limited knowledge of the crosstalk between rumen epithelium-attached bacteria and the host’s cells to date. In this study, we integrated the microbiome, metabolome, and single-cell transcriptome of rumen epithelial tissue to explore the differences in microbiota-host crosstalk between the NB and AD cattle.

In newborn calves, the immune cells were dominated by innate immune cells (dendritic cells), while in adult cattle (as also shown in our multi-tissue study [19]), immune cells were dominated by adaptive immune cells (Th17 cells). This difference between newborns and adults agreed with the results of a previous study that reported Langerhans cells (a dendritic cell type) were present in relative large numbers and T cells occurred at low frequency in the rumen mucosa of the sheep fetus, while T cells (mainly CD4^+^ T cell subsets) were present in relative large numbers in the adult rumen mucosa [52]. Dendritic cells play an important role in maintaining gastrointestinal homeostasis and priming the adaptive immune system by processing and presenting antigens to T cells [53]. There was a large number of dendritic cells presented in newborn rumen tissues, suggesting that the newborn ruminal environment is primed for T cell recruitment and memory generation. Josefsen et al. [52] inferred that the prevalence of T cells in the adult rumen mucosa may be influenced by antigen leakage through the epithelium. The results of the present study may be attributed to this reason that the rumen of adult cattle had experienced changes in diet (from milk to pellets and hay) and environment, and continuous exposure to feed, environmental antigens, and a variety of symbiotic bacteria may make the rumen the main site of pathogen infection. Previous studies reported that the commensal microbe, segmented filamentous bacterium (SFB), induced the generation of homeostatic Th17 cells that reside at mucosal surfaces where they protect host from pathogenic bacteria in the rodent gastrointestinal tract [54, 55]. However, inconsistent with this, the present study showed a high percentage of Th17 cells in AD rumen compared with NB rumen but no detection of SFB in both groups. This may be due to species specificity of the bacteria-dependent accumulation of Th17 cells. *Bifidobacterium adolescentis*, the analogously functioning microbes of SFB, that could induce Th17 cells in the murine intestine was found in humans [56]. Therefore, further studies are needed to identify bacterial species from the bovine rumen microbiota capable of inducing Th17 cells.

Single-cell transcriptomics offers the opportunity to compare cell types across species. The rumen epithelium is composed of stratified squamous epithelium, while the human stomach is composed of columnar epithelium [16, 57]. Due to the different anatomical structures of epithelium in the bovine rumen and the human stomach, it is reasonable that bovine rumen epithelial cells are not similar to that of human stomach. A previous study reported the conserved gene expression patterns between skin and rumen based on traditional molecular biology techniques (bulk RNA sequencing) [39]. Although anatomically similar to rumen epithelium, the epithelial cells of human skin (also composed of stratified squamous epithelium) were not similar to those of bovine rumen at single-cell resolution in this study, which suggested that the species difference overrides the cell-type similarity in orthologous gene expression. We observed that the *IGFBP3*^*+*^ EC appeared only in newborn calves considered to be functionally monogastric animals. The *IGFBP3* encodes a protein that is known to inhibit the role of IGFs in cell proliferation [58]. This indicates that the disappearance of *IGFBP3*^*+*^ EC may facilitate the rumen epithelium development. Moreover, fibroblasts and vascular endothelial cells were more abundant in the newborn rumen, which may contribute to the rapidly postnatal development of rumen epithelial tissue of newborn calves.

In this study, noticeable changes in the mucosal microbiota were observed between the NB and AD groups, which was reflected by alpha diversity indices (significantly increased in the AD group) and a few genera shared between groups. There were 95 and 141 genera with relative abundance greater than 0.01% in the NB and AD groups, respectively, but only 25 were shared between the two groups, and 10 and 22 genera with relative abundance greater than 1% were found in the NB and AD groups, respectively, but only 1 of them was shared between the two groups. Similar observations have been reported for the goat ruminal epithelial bacteria [15] and the bovine rumen bacterial community [59]. Jiao et al. [15] reported that the alpha diversity indices of goat ruminal epithelial bacteria increased with age after birth and each age group had its distinct epithelial microbiota. Jimi et al. [59] also reported that the diversity of the bacterial community in the bovine rumen fluid increased with age and the similarity of bacteria between 1~3-day-old and other age groups was all very low, along with only a few shared genera. The genera *Escherichia-Shigella*, *Streptococcus*, and *Bacteroides* were dominant in NB rumen tissues, which was similar to the results of a previous study that reported the *Escherichia-Shigella*, *Streptococcus*, and *Bacteroides* were dominant at early after birth (0~28 days), but noticeably decreased at 42 or 70 days after birth in the goat rumen epithelium [15]. The genera *Escherichia-Shigella* and *Streptococcus* comprise facultatively anaerobic bacteria, which could create the reduced environment that is required for anaerobic microbes [15, 60, 61]. The predominant genera in adult rumen epithelium mainly included *Butyrivibrio*, *Prevotella*, *Campylobacter*, and *Desulfobulbus*, which were consistent with the results of previous investigations on rumen mucosa of dairy cows [62]. The *Butyrivibrio* genus includes the major known cellulolytic species, and the *Prevotella* genus encompasses a wide array of species that are capable of utilizing different substrates (starches, other non-cellulosic polysaccharides, and simple sugars) [63, 64]. However, little is known about how these genera interacted with the host, which remains to be elucidated.

The most significant transcriptional differences between newborn and adult ruminants were observed in the rumen by comprehensively characterizing transcription of the entire gastrointestinal tract [9]. In this study, compared with the adult rumen, the upregulated DEGs of many epithelial cell subtypes in the newborn rumen were mainly enriched in GO terms related to cell proliferation and development. A previous study found that the higher relative abundance of *Megasphaera* was involved in promoting rumen epithelial growth of calves [65]. Corresponding to this, the present study also showed a higher relative abundance of *Megasphaera* in the rumen epithelium of newborn calves compared with AD ones. *Megasphaera* can convert lactate and glucose into butyrate that is responsible for rumen epithelial proliferation and development [66, 67]. A previous study also suggested that microbes may be involved in the early development of rumen [10], even though the molecular mechanisms behind the development of rumen tissue have been considered ontogenic [68]. These results indicate a potential role of bacteria-driven regulation in the transcriptional activity of rumen epithelial cells; however, further studies are needed to reveal the behind mechanism.

A previous study reported that a co-expressed module gene set involved in “tissue metabolism-related” functions and related to “respiratory electron transport chain” was positively correlated with age of calves and was related to the relative abundance of bacterial genera in the rumen [10]. In the present study, we found that the rumen epithelial cell subtypes had higher metabolism-related functions as well as the oxidation-reduction process at adult stages, which indicates maintaining ROS balance will be very important for the physiological function of cells. A recent study identified a core rumen epithelial microbiota in cattle including the sulfate-reducing bacteria *Desulfovibrio* [11]. The *Desulfovibrio* genus converts sulfate to hydrogen sulfide and plays essential roles in reducing ROS [69, 70]. Our study found the *Desulfovibrio* had a higher relative abundance at the adult stage compared with the newborn stage of the rumen. The relative abundance of *Desulfovibrio* was positively correlated with concentrations of pyridoxal. Genomic analysis of rumen-associated *Desulfovibrio* also revealed that it contains genes that are involved in the pyridoxal biosynthesis [71]. The pyridoxal, as an electron donor for sulfate reduction, not only was oxidized by *Desulfovibrio* to reduce ROS [51] but also prevents host tissue from oxidative stress [72, 73]. Compared with the NB group, adult rumen epithelial cell subtypes showed a higher ability of vitamin B6 (pyridoxal) binding, which suggests that they may uptake more vitamin B6 or participate in B6-mediated signaling pathways. Zhang et al. [74] found that the relative abundance and copy number of *Desulfovibrio* in the rumen was significantly increased after supplementing antioxidants (e.g., resveratrol) in dairy cattle, suggesting that *Desulfovibrio* may be involved in the oxidation-reduction process in the rumen. We also noted that the betaine had the highest co-occurrence probability with the *Desulfovibrio* genus*.* Previous studies reported that the *Desulfovibrio* genus can synthesize and uptake betaine to regulate osmotic pressure for adapting to environmental stress (e.g., high salinity) [75, 76]. Betaine, also as a methyl donor, is involved in regulating the remethylation of homocysteine to methionine, and this reaction is catalyzed by betaine-homocysteine methyltransferase (BHMT) [77]. A recent study showed that rumen-protected betaine supplementation can help improve milk yield and milk protein yield in dairy cows, which may be attributed to sparing methionine from being used as a methyl donor and improving the incorporation of methionine into milk protein [78]. However, no significant difference in the expression level of the *BHMT* gene between cell types of calves and adult cattle was found in this study. Therefore, the mechanisms underlying how the *Desulfovibrio* interact with the rumen epithelial cell subtypes remain to be elucidated.

In addition to these notable findings, there are some limitations in the current study, which will require additional work and new tools to address. Although previous studies reported that a small sample size should be sufficient for the scRNA-seq [79–81], microbiome, and metabolome analysis [15, 82–84], other potential associations between the rumen mucosal bacteria taxa and host cells may not be observed due to the limited number of calves (3 animals per group) used in this study. And although we have enabled deeper and more detailed insights into aspects of the rumen epithelium and its microbiota, these associations mainly represent snapshots in time and space. Future studies to collect and analyze samples with larger sample sizes and more time points may be of great importance to completely understand the host-microbe interactions in the rumen.

## Conclusions

**Fig. 6:**
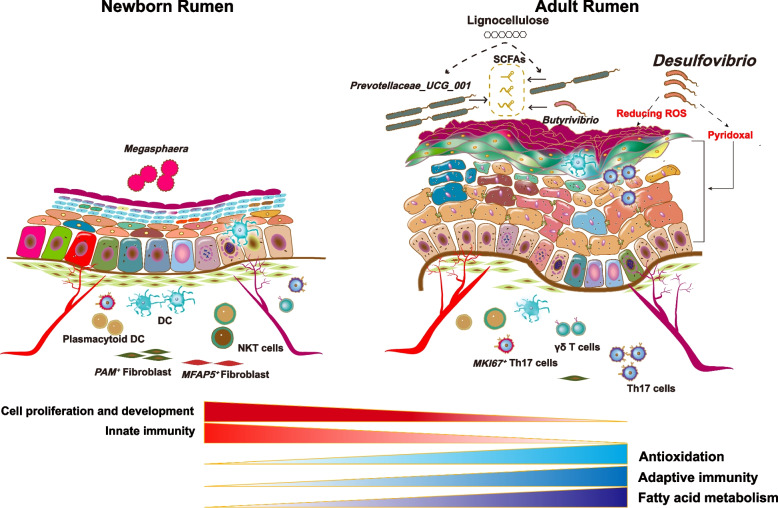
Schematic model summarizing the different epithelial microbiota, cell types, and their interactions between NB and AD rumen. NB, newborn; AD, adult

In summary, we delineated the dynamics of cell-type composition and cellular functions, the relative abundance of the mucosal bacteria, and the potential microbiota-host crosstalk between newborn and adult rumen tissues (Fig. 6). Our integrated analysis of the microbiome, metabolome, and single-cell transcriptome of rumen epithelial tissue provides novel and fundamental insights into the functional completeness of the rumen and may guide future precision feeding in the livestock industry.

## Methods

### Animals

All the animal experimental protocols were conducted in compliance with the Zhejiang University Animal Care guidelines and approved by the Animal Care Committee at Zhejiang University (Hangzhou, China). Three adult Holstein dairy cattle with similar age (3.2~4.4 years old), parity (2~3), milk production (18~22 kg/day), and days in milk (181~200 days) were housed in an open barn and fed the same diet. Three newborn calves (1 day after birth) with similar body weight were transferred to a surgery room soon after birth to collect rumen tissue samples. Epithelial microbiome, metabolome, and single-cell transcriptome analysis were conducted in the same animals.

### Rumen epithelial tissues acquisition

All animals were humanely euthanized in a surgery room to collect samples from the ventral rumen sac. Samples were rinsed in Dulbecco’s phosphate-buffered saline and quickly transferred into cold magnetic activated cell sorting (MACS) tissue storage solution (Miltenyi Biotec, Bergisch Gladbach, Germany) and sent back to the laboratory immediately for single-cell suspension preparation. Samples used for epithelial microbiome and metabolome analysis were snap-frozen in liquid nitrogen and stored at −80 °C.

### Rumen single-cell suspension preparation

After sending back to the laboratory within the cold MACS Tissue Storage Solution, the rumen tissues isolated from adult and newborn cattle were stripped of the outer muscle layers, and then minced into 10 × 0.5mm^2^ pieces on ice with scissors. The tissues were incubated in a 37°C water bath with 20 mM EDTA for 30 min, and then were rinsed with Dulbecco’s phosphate-buffered saline and chopped into 1 mm pieces. Tissue pieces were transferred to a 15-mL centrifuge tube and re-suspended with 0.25% Trypsin-EDTA (Cat# 25200056, Gibco). After incubated in a 37°C water bath for 5 min, the centrifuge tube containing tissues was inserted into ice for 2 min, and pre-chilled Hank’s balanced salt solution (HBSS) was added to stop the digestion. After centrifuging at 300×*g* for 2 min at 4°C, the supernatant was discarded, and samples were washed twice with cold HBSS and then re-suspended with dissociation enzymes. Samples were treated with enzymes (1.5mg/ml collagenase I, 1.5ml/ml collagenase IV, 1.5mg/ml dispase, 100U/ml hyaluronidase, and 50U/ml DNase I) for 30 min at 37°C. The digestion was stopped by adding 10% of fetal bovine serum, followed by a filtration step through a 70- and 30-μm SmartStrainer (Miltenyi Biotec, Bergisch Gladbach, Germany). Samples were centrifuged at 300×*g* for 5 min at 4°C, and then re-suspended in 2 mL of HBSS. Dissociated cells were centrifuged at 300×*g* for 5 min at 4°C, washed twice with 1×PBS with 0.04% BSA, centrifuged at 300×*g* for 5 min at 4°C, and re-suspended in 1×PBS with 0.04% BSA.

Viability for single-cell suspension was assessed via trypan blue using a Countess II Automated Cell Counter. If viability for samples were low, the MACS Dead Cell Remove Kit (Miltenyi Biotec, Bergisch Gladbach, Germany) was used to remove dead cells following the manufacturer’s recommendations. Finally, cells were diluted to a concentration of 700–1200 cells/μl with 1× PBS with 0.04% BSA for 10× Genomics sequencing.

### cDNA library preparation and sequencing

The 10× Genomics Chromium machine was used for single-cell capture, and the library preparation was performed using the Chromium Single Cell 3′ Reagent Kits v3. A single-cell library was generated separately for each animal. After checking the quality using the Agilent Bioanalyzer High Sensitivity chip, the libraries were sequenced on NovaSeq 6000 platform in a 150-bp pair-ended manner.

### Rumen scRNA-seq data analysis

Sequencing results by the NovaSeq 6000 sequencing system were demultiplexed and converted to FASTQ format using Illumina bcl2fastq software. Sample demultiplexing, barcode processing, and single-cell 3′ gene counting was calculated using the Cell Ranger v3.1.0, and scRNA-seq data were aligned to the ARS-UCD1.2 cattle reference genome. The Seurat [85] (version 4.0.3) was used for dimensional reduction, clustering, and analysis of scRNA-seq data. Cells with less than 500 detected genes were considered low quality or empty [22, 86], and cells with more than 4000 detected genes were considered potential doublets [21]. Therefore, for each dataset, we filtered low-quality cells with <500 and >4000 measured genes, UMI counts higher than 50,000, and a mitochondrial gene ratio of higher than 40%. The DoubletFinder [87] package (version 2.0.3) was further used to remove doublets.

For the rumen single-cell atlas, individual newborn and adult Seurat objects were firstly merged into an object. We performed batch correction using Harmony [88] for data integration between samples. The merged object was performed using the NormalizeData, FindVariableFeatures, ScaleData, RunPCA, RunHarmony, FindNeighbors, FindClusters, and RunUMAP functions implemented in the Seurat and Harmony packages. We used the top 30 harmony dimensions and a resolution parameter set to 0.1 for cell clustering. Next, the “FindAllMarkers” function in Seurat was used to determine the marker genes (|‘avg_logFC’| > 0.25 and ‘p_val_adj’ < 0.05) for each cell cluster.

To generate immune cell atlas and non-immune cell atlas in the rumen, we first performed cluster analysis with the top 20 principal components and a resolution of 0.8 for each aforementioned filtered rumen dataset. Cell clusters marked by the immune cell canonical marker gene *PTPRC* were selected and then used for integration to create an immune cell atlas. In this step, the *PTPRC*^*+*^ cell cluster was not found in the Rumen_NB_1 sample (one of the single-cell datasets of calves), which may be due to the small number of immune cells (only 18 immune cells were found in this sample in the above rumen single-cell atlas). A previous study reported that the sufficiently rare cell type (smaller than about 30 cells) will be algorithmically grouped with a more populous cell type because entire cell clusters, rather than individual cells, were annotated in each tissue [89]. Therefore, there were no cells from the Rumen_NB_1 sample in the immune cell atlas. All individual datasets without *PTPRC*^*+*^ cells were then used for integration to create a non-immune cell atlas. Individual *PTPRC*^+^ and *PTPRC*^−^ Seurat objects were merged separately into immune cell and non-immune cell objects, respectively. Each of these merged objects was performed using dimensional reduction, batch-effect correction, and clustering. We performed cluster analysis with the top 50 and 50 harmony dimensions, a resolution of 0.2 and 0.8 for the merged immune and non-immune cell datasets, respectively. Finally, the “FindAllMarkers” function was used to determine the marker genes for each cell cluster. Specifically, at each of the following steps, non-relevant cell types were filtered followed by recalculation of dimensional reduction, batch-effect correction, and clustering. The related marker genes for each cell cluster of immune and non-immune cell atlas are available in the Additional file 2: Table S2 and Table S3, respectively.

### Cell-type composition variation analysis

The numbers of cells of each immune cell type in the different groups (NB and AD) were counted and divided by the total number of immune cells in the same group to calculate the percentage of a given immune cell type for each group. The Log_2_FC between the AD and NB groups was then calculated to identify the cell types altered. The |Log_2_FC| > 0.5 is considered statistically significant according to the published study [81], which means the cell types were significantly altered between the AD and NB groups.

### Cross-species comparison

We downloaded a single-cell RNA-seq dataset of the human stomach from GSE134355 (7 samples) [90] in the Gene Expression Omnibus database and a single-cell RNA-seq dataset of human skin from HRA000395 (3 samples; HRI077736, HRI077737, and HRI077738) [91] in the Genome Sequence Archive database. Firstly, we performed the cell clustering analysis with a resolution parameter setting of 0.8 and 1.0 for the human stomach and human skin, respectively. Next, cell clusters marked by the epithelial cell canonical marker genes were selected in the human stomach and the skin dataset, and the rumen epithelial cell clusters from the non-immune cell atlas were also selected. Finally, the expression matrices (raw counts) of epithelial cell clusters of the two species were merged (cattle rumen and human stomach; cattle rumen and human skin) and orthologous genes were extracted from the data to enable cross-species analysis. The merged expression matrices were used to perform the MetaNeighbour [92] analysis with default parameters. The mean AUROC scores were obtained from MetaNeighbour, and if the AUROC scores between cell types were higher than 0.9, they were considered to be similar or conserved.

### Differential gene expression analysis

We used the “FindMarkers” function in Seurat to identify DEGs for each cell type between NB and AD groups. The normalized read count that was obtained by executing “NormalizeData” and “ScaleData” functions in Seurat was used for the differential gene expression analysis. And the log fold change (Log_2_FC) and *p*-value of each DEG were calculated by using the Wilcoxon rank-sum test as implemented in the “FindMarkers” function. The *p*-value adjustment was performed using Bonferroni correction based on the total number of genes in the dataset. The cell types with fewer than 50 cells in the NB or AD groups were filtered before executing the differential gene expression analysis. Only genes expressed in more than 15% of the cells in the specific cell type were considered. DEGs between the NB and AD groups were identified to generate upregulated and downregulated DEG datasets (|logFC| > 0.5, adjusted *p*-value < 0.05) for each cell type.

### GO term analysis

GO term enrichment analysis was performed using the function enrichGO in clusterProfiler R package [93] based on the dataset “org.Bt.eg.db.” Dot plot of representative GO terms based on the upregulated and downregulated DEGs of cell type were generated with ggplot2 [94].

### 16S rRNA gene sequencing and analysis

Total DNA of the epithelial microbial community was extracted from each rumen epithelial tissue using the E.Z.N.A.® DNA Kit (Omega Bio-Tek, Norcross, GA, USA) according to the manufacturer’s instructions. The qualities and quantities of the DNA samples were determined with a NanoDrop 2000 UV-vis spectrophotometer (Thermo Scientific, Wilmington, USA). The hypervariable region V3-V4 of the bacterial 16S rRNA gene was amplified with primer pairs 338F (5′-ACTCCTACGGGAGGCAGCAG-3′) and 806R (5′-GGACTACHVGGGTWTCTAAT-3′). PCRs were performed with the following program: 95°C for 3 min; 28 cycles (for region V3-V4 of 16S rRNA gene); followed by 72°C for 10 min. PCR products were extracted from 2% agarose gel and purified using the AxyPrep DNA Gel Extraction Kit (Axygen Biosciences, Union City, CA, USA) according to the manufacturer’s instruction and quantified using Quantus™ Fluorometer (Promega, USA). Amplicon sequencing was performed on an Illumina MiSeq platform (Illumina, San Diego, USA) using the paired-end 2×300-bp protocol.

The paired-end reads were merged using FLASH (version 1.2.11) [95]. The 16S rRNA gene sequencing analysis was performed using the QIIME2 [96]. Reads were truncated at the first instance of a quality score less than 20, and then reads with a length less than 50bp or containing N base were removed. In the DADA2 plugin [97], the “filterAndTrim” function with the parameters “MaxEE” setting to 2 and the “truncQ” setting to 0 was used to perform further quality control and chimera was removed using the “consensus” method of the “removeBimeraDenovo” function with the default parameters, and finally, an ASV feature table was produced. The ASV feature table was used for taxonomic identification using a Naiv̈e Bayes classifier trained on the Silva database (Release138, http://www.arb-silva.de) and clustering at 99%. The Shannon and Chao1 indexes of ASVs were obtained in QIIME2. Linear discriminant analysis (LDA) effect size (LEfSe) was used to identify bacterial taxa that were significantly (LDA > 3.5, *p* < 0.05) enriched in NB or AD rumen tissues. The data used for the bacterial composition analysis and the LEfSe analysis were based on the ASVs that were merged to the genus level.

### Metabolomics analysis of rumen epithelial tissues

In the current study, the metabolomics analysis experiment on the newborn rumen tissue was performed at the same time as and using the same batch of reagents as that for the adult rumen tissue in our other study [19]. Specifically, rumen epithelial tissues were homogenized with 1000 μl of ice-cold methanol/water (70%, v/v) and cold steel balls for 3 min at 30 Hz. The tissues were whirled for 1 min without steel balls, and then centrifuged at 4°C, 12,000 rpm for 10 min after 15 min standing. The supernatant was collected for LC-MS/MS analysis.

To identify and quantify as many metabolites as possible in rumen tissues, firstly, all sample extracts are mixed in equal amounts for LC-QTOF-MS/MS experiment to detect metabolites based on the MWDB database built by standard material and public database MHK (including Metlin, HMDB, KEGG database information, secondary spectrum, retention time). Then the information of identified metabolites including multiple ion pair information and retention time is combined with the MWDB database and the accurate quantification information of samples was obtained by Q-Trap. Next, ESI-Q TRAP-MS/MS analysis was performed using the supernatant of each sample. The ESI source operation parameters were as follows: source temperature 500°C; ion spray voltage 5500 V (positive), −4500 V (negative); ion source gas I, gas II, curtain gas were set at 50, 50, and 25 psi, respectively; the collision gas was high. Instrument tuning and mass calibration were performed with 10 and 100 μmol/l polypropylene glycol solutions in QQQ and LIT modes, respectively. A specific set of MRM transitions were monitored for each period according to the metabolites eluted within this period.

The OPLS-DA (orthogonal projections to latent structures discriminant analysis) was performed for the identification of the significantly different metabolites between NB and AD groups. Different metabolites were determined by VIP ≥ 1 and absolute of Log_2_FC ≥ 1. In order to avoid overfitting, a permutation test (200 permutations) was performed.

### Microbe–metabolite vectors (mmvec) neural network analysis

To predict the co-occurrence probabilities between microbes at the genus level and metabolites, the mmvec neural networks analysis was applied [47]. According to the analysis pipeline, the “paired-omics” function with the parameters “epochs” setting to 100 and the “learning-rate” setting to 1e−3 was performed to estimate the conditional probability that each metabolite is present given the presence of specific microbes based on the microbial sequence counts and the metabolite relative concentration. Only microbes at the genus level that appear in at least 2 samples were included in the mmvec neural networks analysis.

### Gene set scoring analysis

The genes of the “vitamin B6 binding” gene set are listed in Additional file 2: Table S9. The signature score of each gene set in each cell type from the NB or AD group was calculated using the AddModuleScore function in the Seurat R package. The differences in the signature scores between two groups of cells were evaluated by a two-sided Wilcoxon rank-sum test.

### Fluorescence in situ hybridization

The rumen tissues collected from the ventral rumen sac of newborn and adult cattle were fixed in a 4% paraformaldehyde fix solution (Beyotime, Shanghai, China) for 24 h and then embedded in paraffin for sectioning. The slices were deparaffinized, dehydrated, and then boiled in the antigen retrieval solution (Cat#G1202, Servicebio) for 10 min. After cooling, slices were added with proteinase K solution (Cat#G1234, Servicebio) and incubated at 37°C for 20 min. Slices were washed three times with phosphate-buffered saline and then added with hybridization buffer (20% formamide) and incubated at 37°C for 1 h. For bacterial FISH, after removing the hybridization buffer, slices were incubated with hybridization buffer (20% formamide) containing the bacterial probe (1μM) overnight at 37°C, and then slices were washed using 2×Saline sodium citrate (SSC) for 10 min, 1×SSC twice (5 min each time) at 37°C, and 0.5×SSC for 10 min at room temperature, respectively. Nuclei were counterstained with DAPI for 8min in the dark. Images were taken using a Pannoramic DESK scanning microscope and analyzed by Caseviewer (version 2.3). The bacterial probe specific to *Desulfovibrio* used for this study was 5′-FAM-GGTCGCCCCCCGACACCT -FAM-3′ (Cat# SPZL000913, EXONBIO).

### Quantification of bacterial density

Absolute quantitative real-time PCR was performed to determine the bacterial densities for the genera *Enterococcus*, *Butyrivibrio*, *Prevotella*, and *Desulfovibrio* between newborn and adult rumen epithelium. Primers are specific to the genera *Enterococcus*: forward primer (CCCTTATTGTTAGTTGCCATCATT) and reverse primer (ACTCGTTGTACTTCCCATTGT) [98]. Primers specific to the genera *Butyrivibrio*: forward primer (GYGAAGAAGTATTTCGGTAT) and reverse primer (CCAACACCTAGTATTCATC) [99]. Primers are specific to the genera *Prevotella*: forward primer (GGTTCTGAGAGGAAGGTCCCC) and reverse primer (TCCTGCACGCTACTTGGCTG) [12]. Primers specific to the genera *Desulfovibrio*: forward primer (ACCTGCTGGAACTGCAARA (R = G or A)) and reverse primer (GTGGAAGCCCACGCTGTT) [100].

### Statistical analysis

The experimental data were performed using a two-tailed Student’s test in GraphPad Prism software (version 8.0) to compare the differences between NB and AD groups assuming equal variance. *P* < 0.05 is considered statistically significant.

## Supplementary Information

**Additional file 1: Fig. S1.** The changes in the expression (CPM) of fibroblasts marker genes (*COL3A1*, *PAM*, and *MFAP5*) between the newborn and adult rumen tissues from the bulk-seq experiment. **Fig. S2.** Comparison of epithelial cell landscapes of human stomach and cattle rumen. (A) The UMAP maps of the human stomach single-cell data, cells are colored by cell types. (B) The UMAP maps representing the expression of representative marker genes among epithelial cell types of human stomach. (C) Similarity of epithelial cell types between human stomach and cattle rumen. AUROC scores were used to measure the similarity of cell types: red, high correlation; blue and yellow, low correlation. The AUROC scores in the diagonal are meaningless according to the scoring system and are shown as blanks. **Fig. S3.** Comparison of epithelial cell landscapes of human skin and cattle rumen. (A) The UMAP maps of the human skin single-cell data, cells are colored by cell types. (B) The UMAP maps representing the expression of representative marker genes among epithelial cell types of human skin. (C) Similarity of epithelial cell types between human skin and cattle rumen. AUROC scores were used to measure the similarity of cell types: red, high correlation; blue and yellow, low correlation. The AUROC scores in the diagonal are meaningless according to the scoring system and are shown as blanks. **Fig. S4.** The rarefaction of the rumen bacteria based on the 16S rRNA in newborn and adult dairy cattle. NB: newborn; AD: adult. **Fig. S5.** The relative abundance of the bacterial taxa at the genus level that were significantly enriched in newborn calves and adult cows.

**Additional file 2: Table S1.** The information of the number of cells for each cell type. **Table S2.** Marker genes for each cell cluster for immune cells. **Table S3.** Marker genes for each cell cluster for non-immune cells. **Table S4.** Marker genes for each cell cluster of human stomach. **Table S5.** Marker genes for each cell cluster of human skin. **Table S6.** Differentially expressed genes and their average read counts in each cell types (adult vs. newborn groups). **Table S7.** The differential metabolites between newborn and adult groups (adult vs newborn). **Table S8.** The microbiota-dependent 38 metabolites with high co-occurrence probabilities (the inferred conditional probabilities >1) with *Desulfovibrio* genus. **Table S9.** Genes in vitamin B6 binding gene set.

## Data Availability

The single-cell RNA sequencing raw data of the adult rumen were previously analyzed in our cross-tissue single-cell transcriptomic landscape study [19] and are available under accession number SRP321626 [101] in the NCBI Sequence Read Archive (SRA) database. The raw single-cell RNA sequencing data of the newborn cattle rumen, and the merged processing files of single-cell RNA sequencing data from the newborn and adult cattle rumen tissues, have been deposited to the Gene Expression Omnibus database under accession number GSE183285 [102]. The raw metabolomic data from the newborn and adult rumen have been deposited to the Open Archive for Miscellaneous Data of National Genomics Data Center (NGDC) under accession number OMIX002037 [103] and also been deposited in the MetaboLights database with the identifier MTBLS6501 [104]. The raw files of the 16S rRNA gene sequencing data of the newborn and adult rumen have been deposited to the SRA database under accession number PRJNA846365 [105]. The processed sequencing data on the expression profiles (CPM) of the fibroblast marker genes in newborn and adult cattle rumen were collected from the GSE74329 [106] and GSE78197 [107], respectively. Other relevant data are available upon request. This study did not generate any unique code.

## Abbreviations

AD: Adult
BC: Basal cells
BC1: Basal cell 1
BC2: Basal cell 2
BC3: Basal cell 3
cg-like SC: Channel-gap-like spinous cells
CPM: Counts per million
DC: Dendritic cells
DEGs: Differentially expressed genes
EC: Epithelial cells
FISH: Fluorescence in situ hybridization
GC: Granule cells
GC1: Granule cell 1
GC2: Granule cell 2
GC3: Granule cell 3
GO: Gene Ontology
HBSS: Hank’s balanced salt solution
MACS: Magnetic activated cell sorting
MC: Mitotic cell
MC1: Mitotic cell 1
MC2: Mitotic cell 2
mmvec: Microbe–metabolite vectors
NB: Newborn
NKT: Natural killer T cells
ROS: Reactive oxygen species
SC: Spinous cells
SC1: Spinous cell 1
SC2: Spinous cell 2
SFB: Segmented filamentous bacterium
scRNA-seq: Single-cell RNA sequencing
VEC: Vascular endothelial cells
VIP: Variable importance in projection.
