# Supplementary material for: Microbiota-host crosstalk in the newborn and adult rumen at single-cell resolution
Source: BMC Biol. 2022 Dec 14;20:280. doi: 10.1186/s12915-022-01490-1 (PMC9749198; doi:10.1186/s12915-022-01490-1)
Supplement: Supplementary file 1 — Additional file 1: Fig. S1. The changes in the expression (CPM) of fibroblasts marker genes (COL3A1, PAM, and MFAP5) between the newborn and adult rumen tissues from the bulk-seq experiment. Fig. S2. Comparison of epithelial cell landscapes of human stomach and cattle rumen. (A) The UMAP maps of the human stomach single-cell data, cells are colored by cell types. (B) The UMAP maps representing the expression of representative marker genes among epithelial cell types of human stomach. (C) Similarity of epithelial cell types between human stomach and cattle rumen. AUROC scores were used to measure the similarity of cell types: red, high correlation; blue and yellow, low correlation. The AUROC scores in the diagonal are meaningless according to the scoring system and are shown as blanks. Fig. S3. Comparison of epithelial cell landscapes of human skin and cattle rumen. (A) The UMAP maps of the human skin single-cell data, cells are colored by cell types. (B) The UMAP maps representing the expression of representative marker genes among epithelial cell types of human skin. (C) Similarity of epithelial cell types between human skin and cattle rumen. AUROC scores were used to measure the similarity of cell types: red, high correlation; blue and yellow, low correlation. The AUROC scores in the diagonal are meaningless according to the scoring system and are shown as blanks. Fig. S4. The rarefaction of the rumen bacteria based on the 16S rRNA in newborn and adult dairy cattle. NB: newborn; AD: adult. Fig. S5. The relative abundance of the bacterial taxa at the genus level that were significantly enriched in newborn calves and adult cows. [file 12915_2022_1490_MOESM1_ESM.pdf]

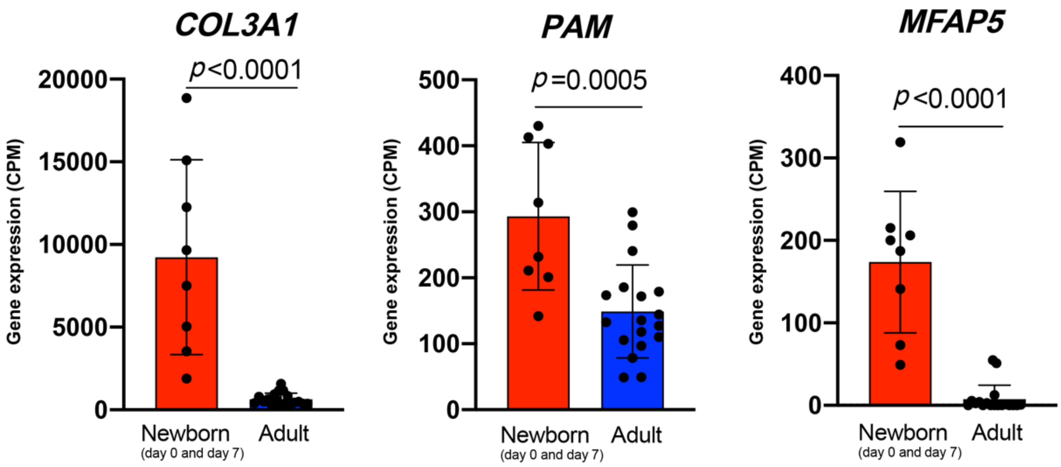

Figure S1. The changes in the expression (CPM) of fibroblasts marker genes (*COL3A1*, *PAM*, and *MFAP5*) between the newborn and adult rumen tissues from the bulk-seq experiment.

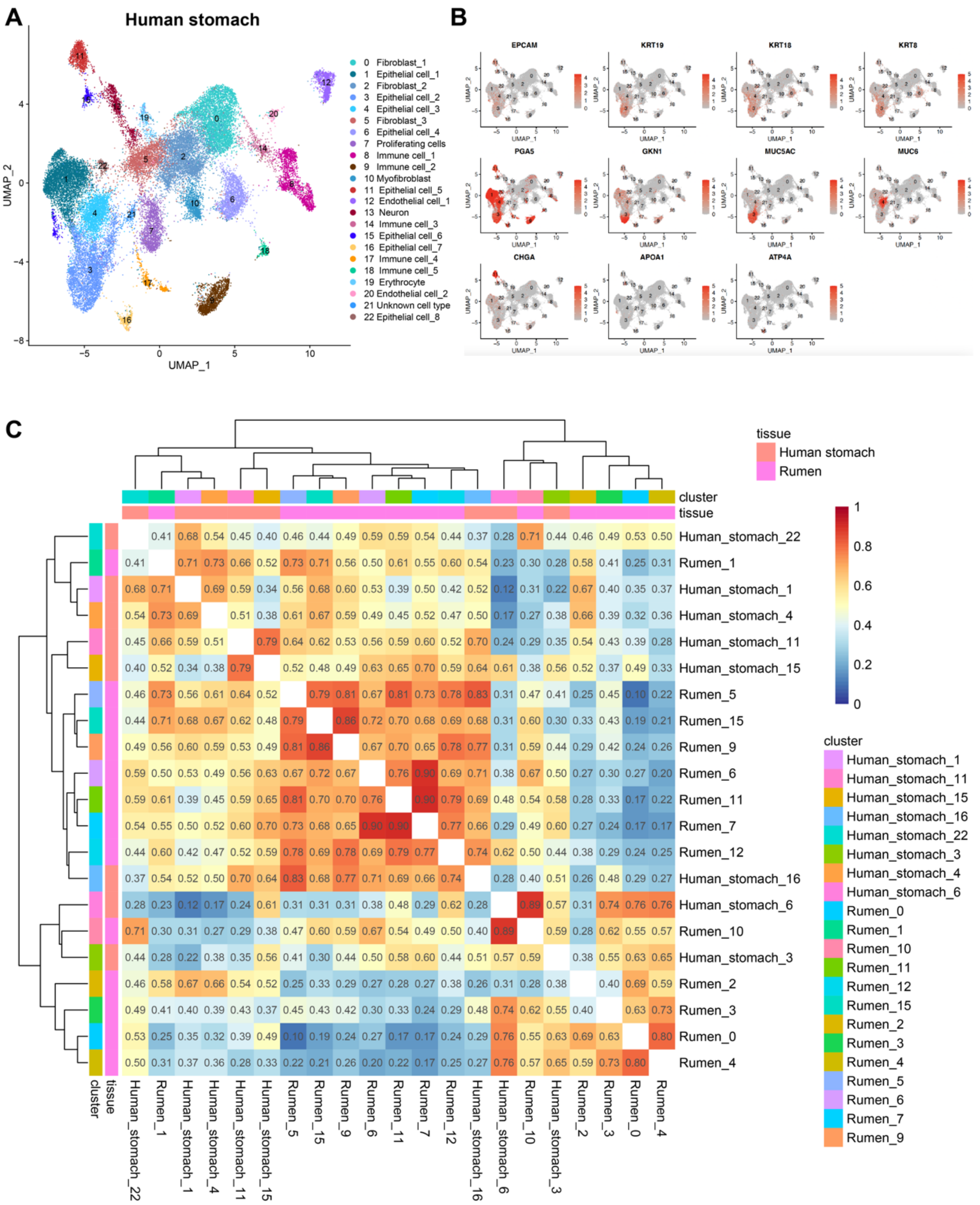

Figure S2. Comparison of epithelial cell landscapes of human stomach and cattle rumen. (**A**) The UMAP maps of the human stomach single-cell data, cells are colored by cell types. (**B**) The UMAP maps representing the expression of representative marker genes among epithelial cell types of human stomach. (**C**) Similarity of epithelial cell types between human stomach and cattle rumen. AUROC scores were used to measure the similarity of cell types: red, high correlation; blue and yellow, low correlation. The AUROC scores in the diagonal are meaningless according to the scoring system and are shown as blanks.

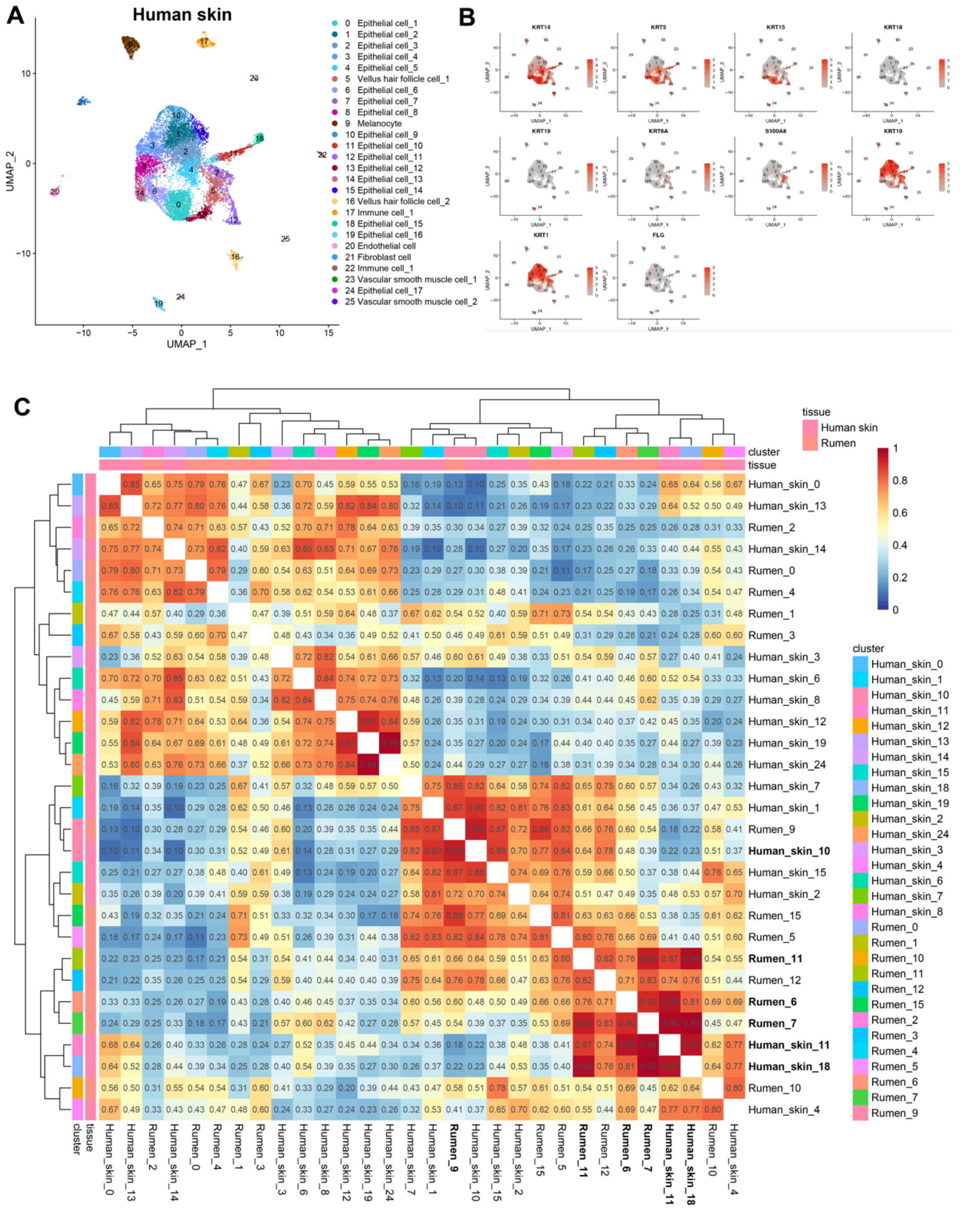

Figure S3. Comparison of epithelial cell landscapes of human skin and cattle rumen. (**A**) The UMAP maps of the human skin single-cell data, cells are colored by cell types. (**B**) The UMAP maps representing the expression of representative marker genes among epithelial cell types of human skin. (**C**) Similarity of epithelial cell types between human skin and cattle rumen. AUROC scores were used to measure the similarity of cell types: red, high correlation; blue and yellow, low correlation. The AUROC scores in the diagonal are meaningless according to the scoring system and are shown as blanks.

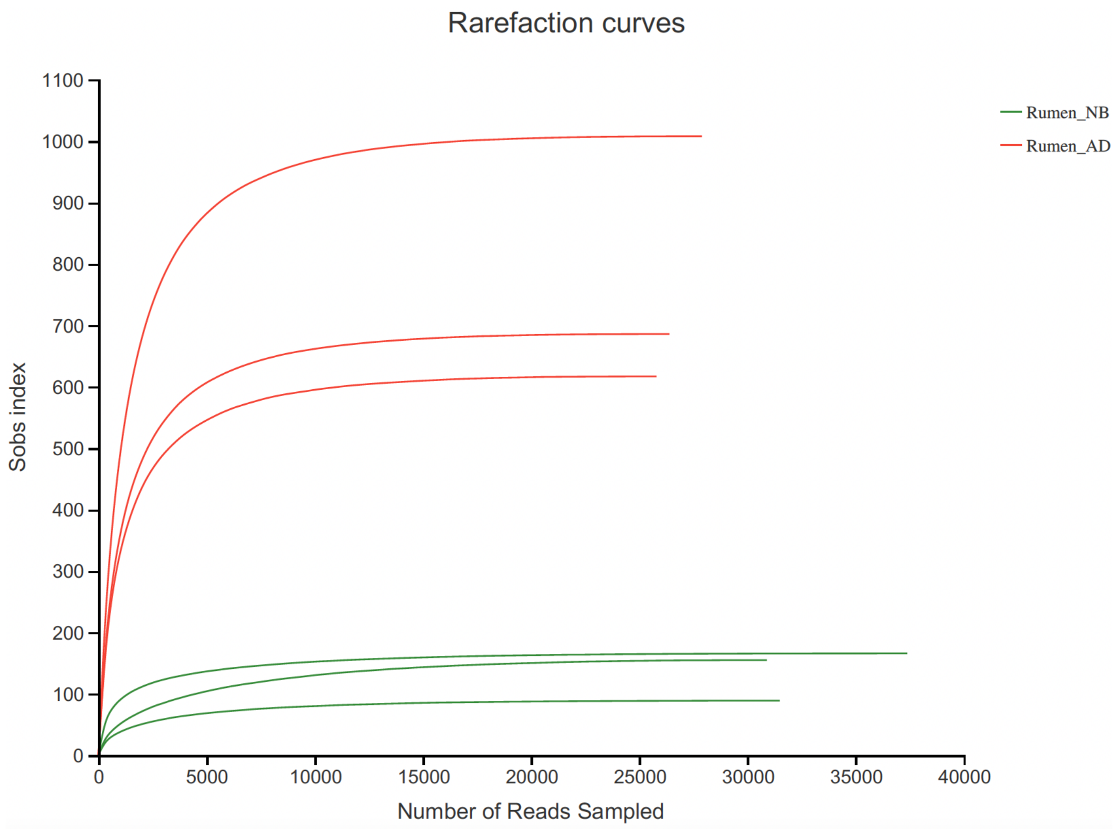

Figure S4. The rarefaction of the rumen bacteria based on the 16S rRNA in newborn and adult dairy cattle. NB: newborn; AD: adult.

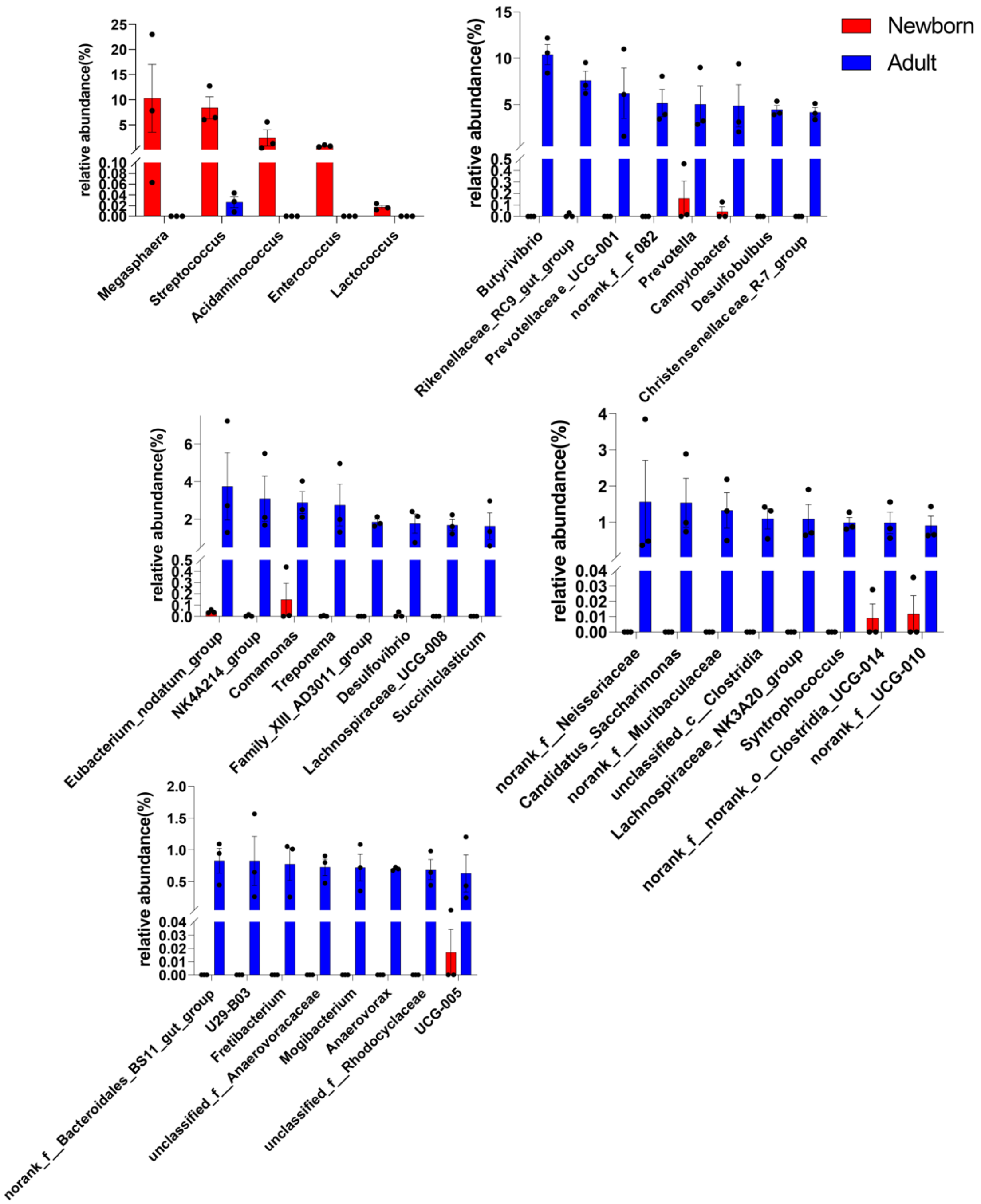

Figure S5. The relative abundance of the bacterial taxa at the genus level that were significantly enriched in newborn calves and adult cows.
